#  Use of a “tablet pole” for the administration of ivermectin for strongyloidiasis in a field study in Ecuador

**DOI:** 10.1186/s40249-023-01054-7

**Published:** 2023-01-28

**Authors:** Dora Buonfrate, Mariella Anselmi, Rosanna Prandi, Monica Marquez, Cristina Mazzi, Antonio Montresor

**Affiliations:** 1 Department of Infectious Tropical diseases and Microbiology, IRCCS Sacro Cuore Don Calabria Hospital, Negrar, Verona Italy; 2 Centro de Epidemiologia Comunitaria y Medicina Tropical (CECOMET), Esmeraldas, Ecuador; 3 Clinical Research Unit, IRCCS Sacro Cuore Don Calabria Hospital, Negrar, Verona Italy; 4 Department of Control of Neglected Tropical Diseases, World Health Organization, Geneva, Switzerland

**Keywords:** Ivermectin, *Strongyloides stercoralis*, Strongyloidiasis, Tablet pole, Preventive chemotherapy, Ecuador

## Abstract

**Background:**

Establishment of efficient control programs for strongyloidiasis, the infection by *Strongyloides stercoralis*, is among the World Health Organization (WHO) targets for 2030. Ivermectin is a drug of choice for strongyloidiasis, but its weight-based administration can be unfeasible in remote areas. We evaluated a WHO tablet pole for administration of ivermectin in school-age children living in remote villages in Ecuador.

**Methods:**

Children were enrolled in 16 villages in Esmeraldas Province of Ecuador, between July 2021 and June 2022. The pole identified four height intervals corresponding to ivermectin doses going from one to four tablets. For each child, we calculated the dose (µg/kg) administered with both weight-based and pole-based administration. Results were classified as follows: optimal dose, acceptable, overdose, underdose. Agreement between the two methods for estimating the number of tablets was assessed with Cohen’s kappa coefficient. Estimations were reported with 95% confidence intervals (*CI*s).

**Results:**

Total of 778 children (47.3% female) were enrolled, with median age of 9.59 years (interquartile range: 7.42‒11.22). Optimal dose was achieved for a higher proportion of children when assessed with weight (37.9%) than with pole (25.7%). Underdose and overdose were more frequent with the pole (8.3% and 19.2% children, respectively) than with the weight-based (3.7% and 6.0%, respectively) administration. Agreement between weight-based and pole-based administration was moderate: 0.56 (95% *CI* 0.51, 0.61). The two methods indicated the same number of tablets in 71.6% (95% *CI* 0.684, 0.748) cases.

**Conclusions:**

In our setting, the tablet pole could be a valid alternative. The tool needs further evaluation in different populations.

**Graphical Abstract:**

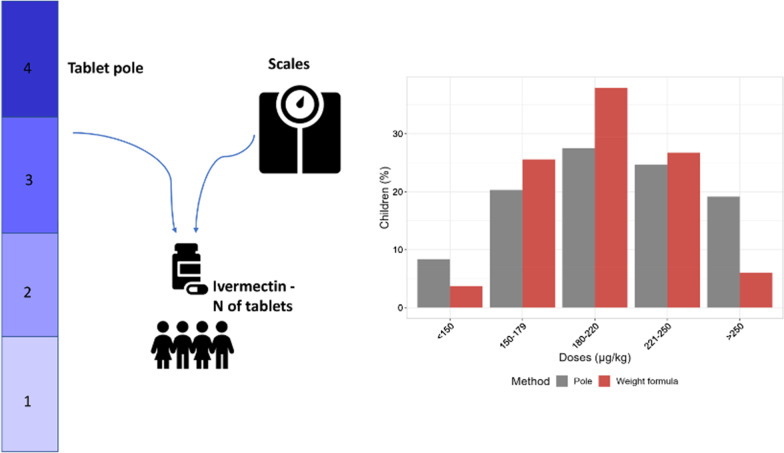

## Background

Strongyloidiasis is a neglected tropical disease (NTD) mainly caused by the soil-transmitted helminth (STH) *Strongyloides stercoralis*, rarely by other *Strongyloides* spp. [1]. According to recent estimates, about 600 million people are infected worldwide, in particular in areas of poor sanitation [2]. More than 50% of infected people with chronic infection report symptoms, pruritus being the main one [3]. In immunosuppressed people, the infection can evolve into a severe condition, hyperinfection or dissemination, which is associated with a high case fatality rate [1].

For the first time in 2021, the World Health Organization (WHO) recommended the implementation of control activities for strongyloidiasis in endemic areas [4]. This has been possible, among other reasons, due to the pre-qualification with the WHO of generic ivermectin products [5], which can ease the access to the drug also in countries where it is not licensed. Currently, ivermectin is donated to endemic countries only in the context of elimination programs for onchocerciasis and lymphatic filariasis (LF) [6].

A single administration of ivermectin demonstrated good efficacy for the treatment of strongyloidiasis [7]. This can favor the integration with control programs for the other STH, which are based on the single administration of albendazole (400 mg) or mebendazole (500 mg) [4, 8]. However, while benzimidazoles are delivered in a fixed dose, ivermectin administration is weight-dependent, recommended at a dose of 200 µg/kg for the treatment of strongyloidiasis [4].

The use of weighing scales can be difficult in the field, and their reliability is often questionable, in particular in the context of mass administration programs. Hence, the use of “tablet poles” has been proposed as an alternative for drugs with weight-dependent administration, e.g., praziquantel and ivermectin [9–11]. The tablet pole is a tool that permits to estimate the number of tablets to be delivered based on height instead of weight. Validation of the pole is needed at country level, in order to evaluate its accuracy for specific populations.

In this work, we report data on the performance of a WHO tablet pole [12] for the administration of ivermectin to school-age children in remote villages of Esmeraldas Province, Ecuador.

## Methods

### Study setting and population

This was a cross-sectional study. School-age children were recruited in 16 villages in Esmeraldas Province, Ecuador, between July 14, 2021 and June 11, 2022. The children were enrolled in the context of a diagnostic study (“ESTRELLA”, registered with Clinical Trials: NCT04999774). The present work is hence a sub-study of the “ESTRELLA” master study.

### Data sources

**Fig. 1:**
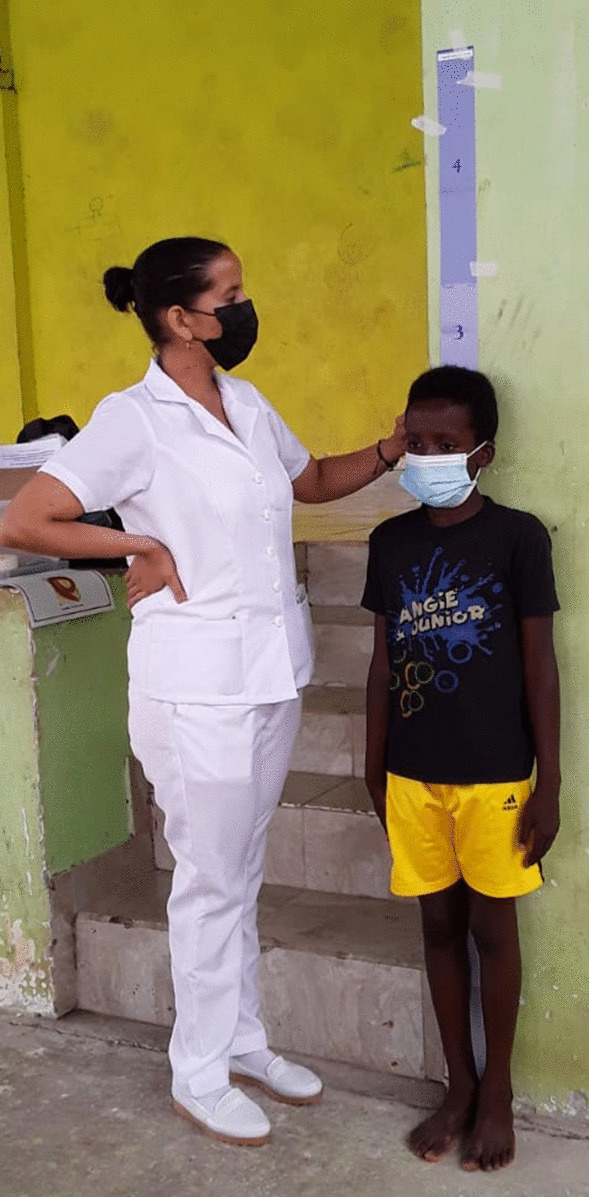
Study staff checking number of tablets to be administered according to the tablet pole

The tablet pole was supplied by the WHO. It identified four height intervals, each one corresponding to a dose of ivermectin going from one to four tablets. The study staff registered height, weight and pole interval of each enrolled child (Fig. 1). InsudPharma & Mundo Sano donated ivermectin (3 mg tablets), which was administered on the weight basis to children with *Strongyloides* infection.

### Variables

Continuous variables (age, weight and height) were summarized with medians and interquartile ranges (IQR). Count variables (i.e. sex and number of tablets) were summarized with absolute and percentage frequencies. For each child we calculated the dose (in µg/kg) administered (rounding to the nearest whole 3 mg tablet) with each method (weight-based and pole-based administration). Results were classified in four categories:

(i) Optimal dose 180‒220 µg/kg (i.e. target dose of 200 µg/kg ± 10%), (ii) acceptable dose 150–250 µg/kg (i.e. within ± 50 µg/kg from the standard dose), (iii) overdose > 250 µg/kg (50 µg/kg more than the standard dose), (iv) underdose < 150 µg/kg (50 µg/kg less than the standard dose).

### Data analysis

Statistical analyses were performed in R* version 4.2.1 [13]. Multivariable linear regression models were used to assess if and how these differences varied over age classes and sex. Model-building strategies included checking for normality of residuals, homogeneity of variance, collinearity and convergence. Then, we calculated the agreement between the methods for estimating the number of tablets obtained through the weight formula and the pole using Cohen’s kappa coefficient. The coefficient values were interpreted as follows: ≤ 0 as indicating no agreement; 0.01–0.20 as none to slight; 0.21–0.40 as fair; 0.41–0.60 as moderate; 0.61–0.80 as substantial; and 0.81–1.00 as almost perfect agreement [14]. The formula for estimating the number of tablets based on weight was (weight × 0.2)/3. Estimations were reported with 95% confidence intervals (*CI*s).

## Results

### Participants

Total of 778 children were enrolled in ESTRELLA, and all of them were included in the present sub-study. Data on weight, height and tablet pole interval were available for all included children.

Median age was 9.59 years (IQR: 7.42‒11.22), 47.3% of the children were female. Median weight and height were 29.0 kg (IQR: 23.0‒36.0) and 136 cm (IQR: 124–145), respectively.

### Main results

**Fig. 2:**
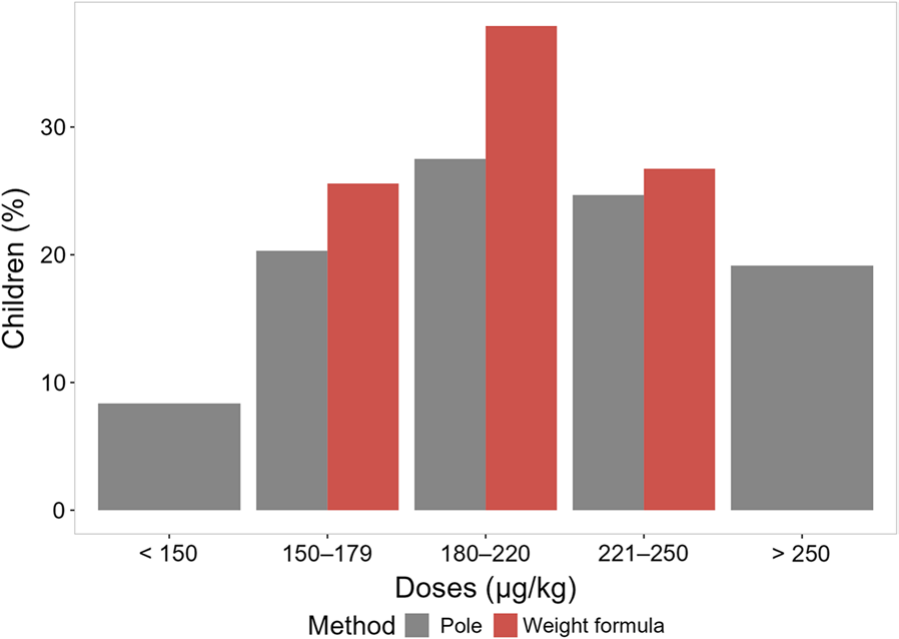
Doses (µg/kg) of ivermectin calculated either by pole or by weight rounding to the nearest whole 3 mg tablet

The proportion of children receiving the optimal dose would be higher with the weight-based approach (37.9%) rather than with the pole-based (27.5%) administration (Fig. 2). With the weight-based calculation, 90.2% children received a dose deemed acceptable, while 72.5% children received an acceptable dose according the dose pole. The proportion of children receiving either an underdose or an overdose would be higher according to the pole (8.3% and 19.2%, respectively) than to the weight (3.7% and 6.0%, respectively).

Both with the pole and the weight assessments, overdosage showed an upward trend with increasing age, as indicated by multivariable linear regression coefficients (respectively: 5.7, 95% *CI* 4.3, 7.0; and 2.3, 95% *CI* 1.2, 3.3). This means that the dose increases by an average 5.7 µg/kg for every year of age with the pole and by 7 µg/kg with the weight. With the pole assessments only, the doses for girls tended to be slightly underestimated compared to those for boys (− 8.0 µg/kg difference, 95% *CI* − 14.3, − 1.7).

The number of tablets to be delivered according to either weight or tablet pole is reported in Table 1. Cohen’s kappa coefficient of agreement between weight-based and pole-based administration was moderate: 0.56 (95% *CI* 0.51, 0.61). The two methods indicated the same number of tablets in 71.6% (95% *CI* 0.684, 0.748) cases. Of note, an individual would be assigned four tablets by pole, while received two by weight; another one would be assigned three tablets by pole, while received six tablets by weight.

**Table 1:** Number of tablets of ivermectin to be administered according to either weight or tablet pole, by age

Age, years	Number of children	Median weight (IQR)	Number of tablets calculated by weight: *n* (%)	Number of tablets calculated by pole: *n* (%)
4	13	18.0 (15.0‒19.0)	1: 13 (100)	1: 13 (100)
5	72	19.0 (17.0‒20.0)	1: 64 (88.9) 2: 8 (11.1)	1: 62 (86.1) 2: 10 (13.9)
6	83	21.0 (19.0‒23.0)	1: 55 (66.3) 2: 27 (32.5) 3: 1 (1.2)	1: 54 (65.1) 2: 28 (33.7) 3: 1 (1.2)
7	73	24.0 (20.0‒25.3)	1: 32 (43.8) 2: 40 (54.8) 3: 1 (1.4)	1: 24 (32.9) 2: 47 (64.4) 3:2 (2.7)
8	79	26.0 (24.0‒30.0)	1: 10 (12.7) 2: 63 (79.7) 3: 5 (6.3) 4: 1 (1.3)	1: 8 (10.1) 2: 69 (87.4) 3: 2 (2.5) 4: 0
9	118	29.0 (26.0‒35.0)	1: 6 (5.1) 2: 91 (77.1) 3: 18 (15.3) 4: 3 (2.5)	1: 5 (4.2) 2: 81 (68.7) 3: 30 (25.5) 4: 2 (1.6)
10	124	32.0 (28.0‒36.0)	1: 2 (1.6) 2: 95 (76.6) 3: 20 (16.2) 4: 7 (5.6)	1: 0 2: 80 (64.5) 3: 42 (33.9) 4: 2 (1.6)
11	115	37.0 (31.2‒43.4)	1: 2 (1.7) 2: 62 (53.9) 3: 44 (38.3) 4: 7 (6.1)	1: 0 2: 36 (31.3) 3: 72 (62.6) 4: 7 (6.1)
12	97	40.0 (34.7‒46.0)	2: 36 (37.1) 3: 48 (49.5) 4: 11 (11.4) 5: 1 (1.0) 6: 1 (1.0)	2: 13 (13.4) 3: 73 (75.3) 4: 11 (11.3) 5: 0 6: 0
13	4	34.5 (32.8‒42.0)	2: 3 (75.0) 3: 1 (25.0) 4: 0	2: 2 (50.0) 3: 1 (25.0) 4: 1 (25.0)

## Discussion

In this work, we evaluated the performance of the WHO tablet pole for the administration of ivermectin in the context of a study carried out in Esmeraldas Province, Ecuador.

Due to the fact that the ivermectin tablets cannot be split into smaller parts, the exact target dose (200 µg/kg) could be provided only to individuals with weight multiple of 15 kg. For this reason, even with the weight-based administration, the optimal dose would not be given to all children, as shown in Fig. 2. However, administration by weight permitted to allocate more children to the optimal and acceptable doses compared to the pole-based administration. Overall, the agreement between the number of tablets calculated by weight and by pole was moderate.

Sex impacted slightly on the performance of the pole, being underdosage more frequent in girls. However, this resulted in a minimal difference in doses for the two sexes, and to us it did not seem to have clinical relevance. Influence of sex on the performance of the pole might have a stronger impact in adults, who have different body compositions.

Overdosage was more frequent for older children, both with the pole and the weight calculation. Indeed, ivermectin has a very favorable safety profile: studies carried out in healthy volunteers with increasing doses, demonstrated safety of doses up to 10 times the standard 200 µg/kg [15]. A systematic review [16] confirmed the high tolerability profile of the drug, and transient eye disturbances were the most frequent adverse events (AEs), mostly reported by individuals with onchocerciasis. Further, the analysis showed that the AEs tended to have an association with increasing levels of microfilaremia rather than ascending doses of ivermectin. Although there are less studies carried out in children, available evidence seems to confirm safety of doses higher than 200 µg/kg also in younger ages. A phase II study [17] evaluated ascending doses, up to 600 µg/kg of ivermectin, in children infected with *Trichuris trichiura*. The drug proved safe and the study demonstrated that the maximum ivermectin concentration and area under curve were twofold lower in children than in adults taking the same weight-dependent dose. The authors hence suggest that more studies should be carried out to establish pediatric dose recommendations.

The results of our study suggest that the main problem with the use of the dose pole might instead be underdosage, that is observed in 8.3% of the children (compared to 3.7% using the scale). Overall, estimated cure rates of strongyloidiasis with a single dose of 200 µg/kg ivermectin are around 86% (95% *CI* 79, 91) [7], and underdosages could lead to lower cure rates, affecting the effectiveness of the intervention and contributing to the emergence of drug resistance. However, it should be considered that in mass treatment campaigns all individuals receive treatment, irrespective of the disease status. This might dilute the impact of underdosage (many individuals who are not infected receive the drug), but further evidence is needed.

Indeed, in light of the complexity of weight check in the field and of the good tolerability of ivermectin, some researchers are evaluating fixed-dose combinations of ivermectin and albendazole [18]. Should this represent an alternative to the current weight-dependent administration, the two methods for ivermectin administration, fixed dose or tablet pole, should be compared to see which one could be more feasible in the context of mass administration.

Previous studies have evaluated the use of height as a proxy for weight for the administration of ivermectin in the context of elimination programs for either onchocerciasis or LF [11, 19]. In the early 1990s, Alexander et al. [19] elaborated four dosing intervals corresponding to different heights, for the administration of ivermectin in the context of a placebo-controlled trial for onchocerciasis. In that study, the target population comprised all individuals aged ≥ 5 years of age, and target dose was 150 µg/kg. Although only 46.5% individuals would have taken the correct dose with the height assessment, underdosage was extremely limited (0.5% cases), and most cases would have deviated from the correct dose by half tablet. Shu et al. [11] confirmed the good performance of the method proposed by Alexander et al. [19] in relation to the small proportion (3.3%) of underdosage, although overdosage was up to 54%. Shu et al. also tested another method of assessment by height, with different dosing levels, which however showed worse performance (21% underdosage).

More recently, Goss et al. [10] compared the WHO ivermectin dosing pole with a dosing method implemented by a modeling approach that, predicting weight by height, aims to estimate the optimal dosing pole thresholds. In this case, the target NTD was LF, and the recommended dose was the same used for strongyloidiasis, 200 µg/kg. Moreover, the dosing intervals were the same considered here, with a maximum of four tablets (although adults were included in their evaluation). They observed 56% individuals who received the same number of tablets by weight and by height. Underdosage was found in 27% individuals assessed with the WHO dosing pole, while using dosing intervals originating from their model, the proportion of underdosage could be reduced to 6%. Underdosage with the WHO pole was more frequent for adult males. Overall, main strength of the dosing method by Goss et al. was adapting the calculation of dosing intervals to the characteristics of specific populations.

Indeed, data should be collected in different geographical areas, to understand whether dosing intervals should vary according to specific characteristics (for instance, different average body mass index) of the population.

The differences in target populations (adults versus school age children), NTD (onchocerciasis, LF and strongyloidiasis) and approach (height measurement, dosing pole and modelling) limit the comparison among studies, but also confirm that the recommendations need to be tailored to the context. For instance, sex might affect slightly the height-based approach in children, while it might have a major impact for adults. For different countries, as suggested also by Goss et al. [10], it could be worth to implement different dosing levels.

Main limitation of our work is that the findings cannot be generalized, and further studies are needed to evaluate the performance of the tablet pole in different contexts.

## Conclusion

The performance of the two methods showed moderate agreement. Although a higher proportion of children would receive the optimal dose based on weight calculation rather than based on the pole, our findings suggest that the latter could be a valid alternative in areas where the scales are not available or not reliable. Further studies are needed to evaluate its use in other countries.

## Data Availability

The raw data of this study  are available in Zenodo: https://zenodo.org/record/7544823#.Y8bGd3ZKiUl.

## Abbreviations

NTD: Neglected tropical diseases
WHO: World Health Organization
STH: Soil-transmitted helminths
LF: Lymphatic filariasis
IQR: Interquartile range
